# Optimizing Peanut (*Arachis hypogaea* L.) Production: Genetic Insights, Climate Adaptation, and Efficient Management Practices: Systematic Review

**DOI:** 10.3390/plants13212988

**Published:** 2024-10-25

**Authors:** Yohannes Gelaye, Huaiyong Luo

**Affiliations:** 1 Oil Crop Research Institute, Chinese Academy of Agricultural Sciences, Wuhan 430062, China; yohanes_gelaye@dmu.edu.et; 2 Department of Horticulture, College of Agriculture and Natural Resources, Debre Markos University, Debre Markos P.O. Box. 269, Amhara, Ethiopia

**Keywords:** climate adaptation, drought-resistant varieties, genetic advancements, integrated pest management, precision farming, sustainable practices

## Abstract

Peanut production plays a crucial role in global food security, particularly in developing countries, where it provides essential nutrition and income. This paper examines the optimization of peanut production through genetic advancements, climate adaptation strategies, and sustainable practices. The primary objective is to increase yields by addressing challenges related to climate change, pests, and resource constraints. Globally, peanut production is hindered by rising temperatures, irregular rainfall, and declining soil quality, impacting both yield and quality. Developing countries, especially in Africa and Asia, face additional challenges, such as limited access to advanced agricultural technologies, inadequate infrastructure, and insufficient support for smallholder farmers. The vital issues include genetic vulnerabilities to pests, climate stress, and inefficient water use. Recent genetic research has provided insights into breeding more resilient, drought-resistant varieties, offering hope for improving yields, despite environmental challenges. The adoption of climate adaptation strategies, precision farming, and integrated pest management is essential for boosting productivity. These, along with optimized irrigation and nutrient management, have significantly impacted peanut production in resource-limited settings. Additionally, drought-resistant varieties have proven crucial, enabling farmers to increase resilience and yields in areas facing climate stress. In conclusion, optimizing peanut production requires continued investment in genetic advancements, infrastructure, and sustainable practices. Future efforts should focus on improving climate adaptation and sustainable farming techniques for long-term success.

## 1. Introduction

Peanuts (*Arachis hypogaea* L.), also known as groundnuts, are believed to have originated in South America, with their domestication first occurring in ancient Peru and Brazil [1]. Over time, peanuts spread to Africa and Asia, eventually becoming a globally cultivated crop. Today, they are a significant agricultural commodity, with China, India, the United States, and Nigeria among the top producers [2,3]. According to the Food and Agricultural Organization (FAO) report of 2002 on Groundnut: Post-Harvest Operations, China and India together are the world’s leading groundnut producers, accounting for nearly 60 percent of the production and 52 percent of the crop area [4]. According to the report provided on peanut production, for the past 25 years, India, China, and the United States have been the leading producers of peanuts, together cultivating approximately 70% of the world’s supply [5]. The GO-TEXAN Market report by the Center for American Studies shows that global peanut production averaged 46.4 million metric tons (MMT) from 2016 to 2020, with China leading at 17 MMT, followed by India, Nigeria, the U.S., and Sudan, which together produced two-thirds of the total [6,7]. A study showed that groundnut yields in Asia and Africa’s Semi-Arid Tropics (SATs) were significantly lower (<900 kg/ha) than the global average (about 1500 kg/ha), with the International Crops Research Institute for the Semi-Arid Tropics (ICRISAT) leading efforts to improve productivity through germplasm research [8].

These regions account for a significant portion of global peanut production, highlighting the crop’s economic importance. Also, the USDA report shows that, in 2019/20, China set a record for peanut imports, surpassing the European Union by over 40% due to slow domestic production and high global stocks, with Senegal, Sudan, and the U.S. supplying nearly 90% of these imports [9].

Peanuts thrive in well-drained sandy loam soils with a pH of 5.9 to 7.0 and require a warm climate with temperatures ranging from 65 to 80 °F (18 to 27 °C) for optimal growth [10]. They benefit from full sun exposure and need about 6 h of direct sunlight daily [11]. These environmental requirements make peanuts well-suited to regions with moderate rainfall and long growing seasons [12,13]. Peanuts offer numerous benefits worldwide, including their nutritional value as a source of protein, healthy fats, and essential vitamins [14]. They play a critical role in food security and agricultural economies, providing livelihoods for millions of farmers [15]. Additionally, peanuts are used in a variety of products, from food items to industrial applications, underscoring their versatility and economic significance [16].

Research and breeding efforts have significantly advanced peanut production, focusing on enhancing yield, disease resistance, and nutritional quality. Innovations in genetic insights have led to the development of more resilient peanut varieties, while ongoing studies aim to address major constraints, such as pest infestations, soil degradation, and climate change [17,18]. Effective breeding strategies and genetic improvements are crucial for sustaining and expanding peanut production [19].

Thus, to address these challenges, optimizing peanut production involves adapting to climate variations and implementing efficient management practices. Also, integrating genetic insights with climate adaptation strategies and adopting best practices in cultivation can enhance productivity and sustainability. Thus, this work aimed to review the optimization of peanut production through genetic advancements, climate adaptation strategies, and efficient management practices.

## 2. Review Methodology

**Figure 1:**
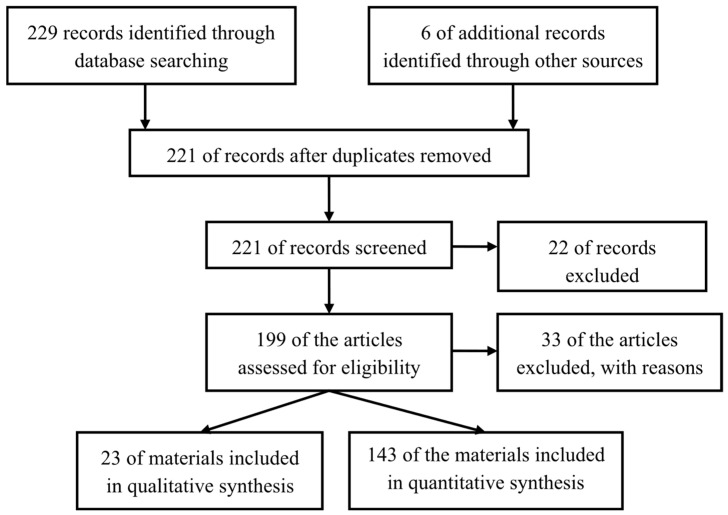
PRISMA flow diagram.

To conduct a comprehensive review on “Optimizing Peanut Production: Genetic Insights, Climate Adaptation, and Efficient Management Practices”, we followed a structured approach to identify, evaluate, and synthesize relevant literature. We searched multiple databases, including Scopus and Web of Science, to ensure comprehensive coverage of peer-reviewed articles and books. Operators were also used to combine index terms and keywords such as “peanut genetics”, “*Arachis hypogaea*”, “climate adaptation”, “drought tolerance”, “peanut management practices”, and “crop optimization”. To ensure the inclusion of recent advancements, we restricted the search to studies mainly published between 2019 and 2024. Therefore, the review materials were selected spanning the years, led by indexes and respective protocols. Moreover, search engines like Google Scholar were used to track the literature and relevant non-indexed studies. Exclusion criteria removed studies focusing solely on unrelated crops, providing insufficient data on peanut production, or lacking peer-reviewed status (Figure 1). Additionally, non-English language articles and those without accessible full texts were excluded. Duplicates were also removed using different techniques, and after eliminating duplicates, the review included studies that met the predefined inclusion criteria. Overall, more than 160 papers were reviewed to write this article, of which 111 articles (66.8%) and 55 articles (35%) were sourced from Scopus and other databases respectively. The majority of the reviewed studies emphasized that genetic insights, climate adaptation strategies, and efficient management practices were vital for strengthening peanut crop resilience, maximizing yields, and fostering sustainable agriculture in response to climate change, ultimately improving food safety and agricultural productivity.

## 3. Advancements in Peanut Genetic Research

Recent advancements in peanut genetics are enhancing crop quality, productivity, and sustainability. Historically a staple providing vital nutrients worldwide, peanuts have faced challenges from diseases, pests, and environmental stresses [19]. The advent of molecular biology and genomic technologies has empowered researchers to develop peanut varieties with superior traits, including enhanced disease resistance, drought tolerance, and nutritional value.

A report on peanuts and their wild relatives revealed that recent cytogenetic studies and molecular phylogenies, combined with new genetic tools, are advancing the efficient use of peanut genetic resources for crop improvement [20].

A two-year (2015 and 2016) field study conducted to characterize the genetic variation and correlation among important yield and quality-related traits of peanut under arid climate conditions at research farm of the College of Agriculture, Bahauddin Zakariya University, Bahadur Sub-Campus Layyah-Pakistan concluded that different peanut genotypes differed in their morphological and yield parameters, pod/seed yield, shelling percentage, oil contents, and fatty acid composition [21]. However, a study on gene expression and deoxyribonucleic acid (DNA) methylation in wild allotetraploid peanut (*A. monticola*) suggests that cytosine–guanine (CG) and cytosine–histone–guanine (CHG) methylation may negatively regulate lipid metabolic genes and transcription factors, influencing oil accumulation [22]. Furthermore, according to the research conducted on gene expression and DNA methylation alteration leading to a high oil content in wild allotetraploid peanut, the expression alteration of lipid metabolic genes with co-expressed transcription factors in wild peanut led to enhanced activity of oil biogenesis and reduced the rate of lipid degradation [22]. Indeed, the programs of plant genetic improvement are dependent upon the diversity and selection of suitable genotypes for an area [23]. A study conducted on basic regulators of sucrose metabolism identified through comprehensive comparative transcriptome analysis in peanuts (China) stated that the key roles of the high expression of SWEETs and enzymes in sucrose synthesis made the genotype ICG 12625 sucrose-rich [24].

In addition, book chapter (I) that documented good agricultural practices for peanut growing and harvesting indicated that the need for oversight extends from the field through shelling, processing, packaging, and delivery to the consumer, with good agricultural practices (GAPs) offering guidelines to producers on minimizing hazards during production and harvest [25]. Moreover, the detailed project report on groundnut oil by the National Institute of Food Technology Entrepreneurship and Management (NIFTEM) in India stated that groundnut oil contained over 80% unsaturated fatty acids, including 41.2% oleic acid and 37.6% linoleic acid, along with 19.9% palmitic, stearic, arachidic, and other unsaturated fatty acids [26]. Correspondingly, research on the genetic dissection of fatty acid components in the Chinese peanut mini-core collection under multi-environments reported that the genetic architecture of fatty acid components in peanut and the new effective diagnostic marker would be useful for the marker-assisted selection of high-oleic peanut breeding [27].

A study conducted to investigate the twenty widely grown Korean genotypes at the seedling stage under control and drought stress conditions and to identify tolerant lines, as well as related traits, concluded that phenotypic characterization or screening of peanut genotypes reiterated the usefulness of uniform evaluation under the control condition to identify promising genetic materials [28]. However, the newly identified tolerant genotypes could be a potential source for conducting multi-location and stability studies to develop drought-tolerant peanut cultivars. Additionally, a review on the physiological responses of groundnut to drought stress and its mitigation suggested that systematically applying knowledge in practice can result in substantial improvements in both yield and yield stability in global groundnut production [29]. These breakthroughs are vital for ensuring food security and meeting the growing global demand for peanuts. One vital milestone has been the sequencing of the complex tetraploid peanut genome, a long-standing challenge to decipher [30,31]. Research on the Chinese peanut mini-core collection using genome-wide association studies identified the genetic basis of yield-related traits and verified a marker to aid in developing high-yield peanut varieties through marker-assisted selection [32].

However, recent advancements in sequencing technologies have allowed scientists to map out the peanut genome more precisely, leading to the identification of crucial genes responsible for desirable traits. This information enables breeders to use marker-assisted selection (MAS) and other tools to accelerate the development of improved peanut varieties, bypassing the slower, more traditional methods of selective breeding. Research using modeling tools to assess climate change impacts on smallholder oilseed yields (including peanut crop) in South Africa suggests that climate change may reduce yields, while management practices could increase emissions, highlighting the need for adaptation strategies that account for both crop impacts and emissions to ensure a comprehensive approach [33].

Additionally, the integration of clustered regularly interspaced short palindromic repeats (CRISPR) and gene-editing technologies has begun to revolutionize peanut genetic research [34]. These tools allow researchers to make targeted changes in the peanut genome, improving traits like oil content, shelf life, and resistance to aflatoxin—a harmful contaminant often found in peanuts [35]. As these technologies continue to evolve, they are expected to play a pivotal role in addressing challenges faced by peanut farmers worldwide, including the effects of climate change and the need for sustainable agriculture practices. Research conducted in Wuhan, China (2018–2019), on the Chinese peanut mini-mini-core collection revealed wide variation in aflatoxin resistance, identifying 14 resistant accessions: 3 for shell infection, 7 for seed infection, and 5 for aflatoxin production resistance [36]. Also, the study identified two peanut genotypes (Zh.h0551 and Zh.h2150) resistant to aflatoxin contamination, along with single-nucleotide polymorphism (SNP) markers, which could aid in breeding for aflatoxin resistance.

## 4. Genetic Markers for Disease Resistance

The use of genetic markers for disease resistance in peanuts has become a vital strategy for breeding varieties that can withstand common diseases like leaf spot, rust, and root-knot nematodes. A study on the peanut variety Zhonghua 6 identified two novel adjacent quantitative trait loci (QTLs) on chromosome B02 governing bacterial wilt resistance, with candidate genes requiring validation through fine mapping or complementation assays [37].

By using genetic markers, breeders can now identify and select peanut plants with the best genetic traits for disease resistance, streamlining the breeding process and improving the crop’s overall resilience [38].

In peanut breeding, MAS is particularly valuable because it allows for the early identification of plants with desirable resistance traits, reducing the time it takes to develop new varieties [39]. This is crucial in combating persistent diseases, especially those caused by fungal pathogens and nematodes, as genetic markers help breeders to efficiently incorporate resistance genes into new peanut varieties [40]. Moreover, peanut breeders are using these genetic markers to stack or “pyramid” resistance genes, which enhances the plant’s ability to resist multiple diseases simultaneously [41]. Pyramiding genes in peanut varieties provides durable, long-lasting disease resistance, reducing farmers’ reliance on pesticides, lowering costs, minimizing environmental impact, and ensuring stable yields in regions with high disease pressures and climate challenges [42]. Genetic markers associated with disease resistance in peanuts enable breeders to identify and track specific resistance genes, making it easier to develop new, disease-resistant varieties (Table 1).

An investigation conducted for analyzing genetic diversity among 59 local groundnut germplasms using Mahalanobis D2 statistical analysis for quantifying the degree of divergence at the genotypic level at the research farm of Rajmata Vijayraje Scindia Agricultural University, Gwalior (M.P.), in India (2020) reported that selected germplasms with diverse characteristics could efficiently be applied in groundnut crop improvement programs [43]. Therefore, the markers are particularly useful in targeting resistance to fungal pathogens and nematodes, which are major threats to peanut crops.

**Table 1:** Genetic markers linked to disease resistance in peanuts.

No.	Genetic Markers	Analytics and Disease Resistance Implications	Ref.
1	AhMLO1	Confers resistance to powdery mildew.	[44]
2	GM630	Associated with resistance to early leaf spot (ELS).	[45]
3	pPGPseq28D09	Identified for groundnut rosette disease (GRD) resistance.	[46]
4	AhRXR	Provides resistance to root-knot nematodes.	[47]
5	AhSNP_163	Linked to rust disease resistance.	[48]
6	GM2009	Marker for resistance to Sclerotinia blight.	[49]
7	AhCDF1	Associated with peanut smut resistance.	[50]
8	AhNBS1	Related to resistance against *Aspergillus flavus* (*A. flavus*) and aflatoxin contamination.	[51]
9	AhCSP1	Linked to resistance against tobacco streak virus (TSV).	[52]
10	SSR_REF180	Used to map genetic variations associated with disease resistance and stress tolerance.	[53]
11	PM36	Used to identify genetic variations linked to traits like disease resistance, particularly in breeding programs.	[54]
12	SSR_HK6	Linked to stem rot resistance.	[55]
13	AhPDR1	Associated with the plant’s defense mechanisms, particularly conferring resistance to diseases and environmental stresses.	[56]
14	GM671	Related to resistance to Cylindrocladium black rot (CBR), a serious fungal disease.	[57]

Peanut cultivars, including, Tifguard, Sedi, and TifNV-High O/L, exhibit a range of adaptations to different climatic and soil conditions, making them valuable for various agricultural contexts worldwide. Each cultivar possesses unique traits, such as nitrogen fixation potential and average yield, which contribute to their suitability for specific regions and farming practices (Table 2). Groundnuts significantly improve soil fertility through nitrogen fixation when intercropped, reducing the need for synthetic fertilizers and boosting companion crop yields [58]. This process supports sustainable farming by preserving soil health and enhancing climate resilience, helping crops to endure climate variability. For African farmers, it offers a cost-effective way to boost productivity and ensure long-term agricultural sustainability [59].

## 5. Enhancing Yield Through Genetic Modification

The use of genetic modification (GM) in agriculture has revolutionized crop production, and peanuts are no exception. Genetic modification offers a powerful approach to improving yield by enhancing traits such as nutrition and nutrient efficiency [19,68]. Although figurative data have not been sufficiently explored, genetic modifications in peanut crops have been shown to increase yields, depending on the specific traits introduced and the environmental conditions [69]. The basic manual on groundnut production highlights that proper cultivation of groundnuts can provide farmers and consumers with a highly nutritious crop and valuable income, while supporting human consumption through raw seeds, peanut butter, oil, and other by-products [70]. With the growing demand for peanuts as a vital food source globally, increasing yields through genetic modification has become a critical focus for scientists and breeders. A book chapter focused on groundnuts states that the groundnut is an important legume nut known for its multifarious uses, including oil production, direct human consumption as food, and also animal consumption in the form of hay, silage, and cake [71]. The peanut guide book prepared by the University of Kentucky (US) College of Agriculture, Food, and Environment Center for Crop Diversification Crop Profile reports that peanuts are an annual herbaceous legume with an indeterminate growth habit [72]. Similarly, the Smallholder Best Practices Guide for Growing Peanuts in Haiti identifies two main peanut types: prostrate, which grow close to the ground, and bunch, which grow upright and produce peanuts near the main root [73]. This strategy not only addresses the challenges of climate change, but also contributes to sustainable agricultural practices.

Hence, one of the key areas where genetic modification has shown promise in peanuts is increasing resistance to diseases [74]. Common peanut diseases, like early leaf spot, late leaf spot, and *A. flavus* (which produces aflatoxin), can drastically reduce yields [75]. Through genetic engineering, scientists can introduce genes from other organisms that provide resistance to these pathogens. For example, antifungal genes have been inserted into peanuts to help the crop fend off devastating fungal infections, leading to healthier plants and higher yields [42]. This reduces the need for chemical fungicides, which can be costly and harmful to the environment. Peanuts are often grown in regions where water is scarce, and drought can significantly impact crop productivity [76]. By introducing genes that enhance the plant’s ability to retain water and tolerate extended periods of drought, genetically modified peanut varieties can maintain their yield potential, even in harsh environmental conditions [68]. This is particularly important as climate change continues to alter weather patterns and increase the frequency of droughts in many peanut-growing areas. Finally, genetic modification can also improve the nutritional content and oil composition of peanuts, which can indirectly enhance yield by making the crop more valuable and marketable [77,78]. Genetic modification can also enhance the levels of essential fatty acids, vitamins, and minerals [79]. Genetically modified peanuts with enhanced features like higher protein contents, better oil quality, and increased pest resistance attract health-conscious consumers and food manufacturers, boosting demand and encouraging farmers to adopt these varieties for better yields and market value [80]. Current research aims to combine yield-enhancing traits with improved nutritional profiles in genetically modified peanuts, ensuring their vital role in feeding the growing global population and promoting agricultural sustainability.

## 6. Impact of Climate Change on Peanut Crops

Climate change poses a significant threat to global agriculture, and peanut crops are particularly vulnerable to its effects. Peanuts, which are primarily grown in tropical and subtropical regions, rely on specific climatic conditions for optimal growth, including well-distributed rainfall, moderate temperatures, and adequate sunlight [81,82]. Research conducted to evaluate the potential benefits of drought and heat tolerance in groundnut for adaptation to climate change in India and West Africa indicates that different combinations of traits will be needed to increase and sustain the productivity of groundnut under climate change at the target sites, and the CROPGRO-Groundnut model can be used for evaluating such traits [83].

Correspondingly, the U.S. Department of Agriculture (USDA) Technical Bulletin 1935 document on Climate Change and Agriculture in the United States: Effects and Adaptation emphasizes that increases in atmospheric carbon dioxide (CO_2_), rising temperatures, and altered precipitation patterns will affect agricultural productivity [84]. Increases in temperature coupled with more variable precipitation will decrease the productivity of crops, including peanut crop, and these effects will outweigh the benefits of increasing carbon dioxide [85]. However, rising temperatures, shifting rainfall patterns, and the increasing frequency of extreme weather events are disrupting these conditions, leading to reduced yields and threatening the livelihoods of millions of farmers who depend on peanut production. A concise guide prepared on groundnut production by A.J. CILLIERS (ARC Grain Crops Institute) Potchefstroom reported that groundnuts must thus be planted as early in the season as possible, when the danger of cold spells has already been reduced [86].

Research conducted to evaluate the effect of climate change factors on processes of crop growth and the development and yield of groundnut indicates that, in spite of the beneficial effect of increased atmospheric CO_2_ concentration, climate change will adversely impact the production and productivity of groundnut grown in subtropical and tropical regions of the world [87]. Thus, the direct and indirect effects of most climate change factors on plant growth and development processes are well understood and have already been incorporated in the CROPGRO-Groundnut model.

Furthermore, a study assessing climate change adaptation strategies among smallholder farmers in Senegal’s semi-arid zone highlighted that a clear understanding of climate indicators within the community may be a crucial factor influencing their decisions regarding crop and livestock management [88]. One of the most direct impacts of climate change on peanut crops is the increase in temperature [87]. Peanuts are sensitive to both heat stress and drought, and higher temperatures can reduce pod formation and affect the quality of the seeds [29]. As global temperatures rise, peanut plants experience shorter growing seasons and reduced water availability, which leads to lower yields [89]. In some regions, extreme heat can cause physiological stress in peanut plants, resulting in poor development, reduced seed size, and lower overall productivity [90]. Changes in rainfall patterns, driven by climate change, are causing prolonged droughts or erratic rainfall in many peanut-growing regions, negatively affecting peanut crops [81]. Drought conditions can lead to water stress, limiting the plant’s ability to develop properly, while excessive rainfall can promote fungal diseases, such as leaf spot and root rot, which can devastate peanut fields [91]. In addition, unpredictable rainfall and the rising incidence of pests and diseases due to climate change make it harder for farmers to manage peanut planting, harvesting, and overall production [92]. Warmer temperatures and higher humidity create favorable conditions for the proliferation of pathogens and insect pests, which can further reduce yields [93]. For example, aflatoxin contamination, a harmful toxin produced by the fungus *A. flavus*, is more likely to occur in stressed peanut plants exposed to high temperatures and drought [94]. To counter the growing environmental pressures from climate change, the peanut industry must adapt with improved management practices, climate-resilient varieties, and sustainable farming techniques.

## 7. Developing Climate-Resilient Peanut Varieties

As climate change continues to disrupt traditional agricultural practices, the development of climate-resilient peanut varieties has become essential for sustaining production. Peanuts are highly sensitive to environmental changes, including temperature fluctuations, erratic rainfall, and increased pressure from pests and diseases [87]. To mitigate these challenges, researchers are focusing on breeding and biotechnology innovations aimed at creating peanut varieties that can thrive in changing climates, ensuring food security and sustainable farming practices for peanut-growing regions. An investigation conducted on the adoption of climate-resilient groundnut varieties increasing agricultural production, consumption, and smallholder commercialization in West Africa concluded that the adoption of climate-resilient groundnut varieties can at least partially decrease production constraints and increase smallholder consumption and commercialization, with implications for agricultural transformation [95]. Also, a review on climate-resilient crops highlighted that progress was slow due to the polygenic nature of key traits and the testing of QTLs and genes under controlled, rather than real, field conditions, and to address this, QTL mapping and genome-wide association studies (GWASs) are used to explore multiple traits and genetic variation [96,97]. Furthermore, a study conducted on morphological characterization and reproductive aspects in genetic variability studies of forage peanut in Brazil (p. 299) reported that the stigma morphology and distinct mode of reproduction present among accessions suggested possible barriers for seed production in some cross-combinations [98].

The basic goal in developing climate-resilient peanuts is enhancing drought and heat tolerance by incorporating traits that improve water-use efficiency and maintain the yield under dry conditions [90,99]. By selecting for genes associated with deeper root systems, increased water retention, and better heat resistance, scientists are working to produce varieties that can maintain productivity in regions increasingly affected by prolonged droughts and higher temperatures [100]. Breeding climate-resilient peanuts also focuses on disease and pest resistance, as rising temperatures and humidity due to climate change promote fungal diseases like leaf spot and aflatoxin contamination [17,101]. Researchers are identifying and integrating genes that offer resistance to these pathogens, either through traditional breeding methods or using molecular techniques, such as MAS and gene editing [102,103]. Efforts to develop peanut varieties resistant to biotic and abiotic stresses aim to reduce crop losses and chemical use, while also enhancing nutritional quality and marketability in response to climate change [104]. Varieties with higher oil content, improved protein quality, and enhanced tolerance to storage conditions are being developed to meet the evolving demands of both consumers and producers [105]. These climate-resilient peanuts not only provide farmers with more stable yields, but also offer higher market value, contributing to economic sustainability. As climate change continues to pose challenges for peanut agriculture, the development of these resilient varieties is critical to ensuring the long-term viability of peanut farming in vulnerable regions [106]. Some peanut varieties have demonstrated resilience to climate change by exhibiting traits like drought tolerance and enhanced nitrogen fixation, making them suitable for challenging environmental conditions (Table 3).

## 8. Soil Management for Optimal Peanut Production

Effective soil management is crucial for maximizing peanut production, as the health and fertility of the soil directly impact the growth, yield, and quality of the crop. Peanuts are sensitive to soil conditions, particularly pH levels, nutrient availability, and soil structure [115]. The Appropriate Technology Transfer for Rural Areas (ATTRA) report on peanuts highlights that organic peanut production focuses on management practices that enhance and sustain soil fertility by optimizing biological activity [116].

Managing these factors is essential to ensure that peanut plants can develop healthy root systems, access essential nutrients, and avoid issues like soil-borne diseases or waterlogging. Also, a peanut production guide from 2020 (Management and Cultural Practices for Peanuts) states that deciding which varieties to plant is very important for achieving the best yield and for marketing the crop, and the varieties differ in their resistance to diseases as well as other characteristics, such as seed size, vine growth, grade, and pod yield [80].

Erect and prostrate peanut crop varieties display distinct growth habits that significantly impact their cultivation and productivity. Erect varieties grow upright, facilitating easier harvesting and improved air circulation, whereas prostrate varieties spread horizontally, effectively suppressing weeds and enhancing soil moisture retention [117]. The selection between these varieties depends on specific agricultural practices and environmental conditions, as each presents unique advantages and challenges concerning yield potential and susceptibility to diseases.

Bulletin (302) prepared on the fertility requirements of runner peanuts in southeastern Alabama summarized that applications of nitrogen fertilizers on runner peanuts did not affect yields or quality [118].

Proper soil management significantly boosts peanut productivity and ensures long-term sustainability [119]. Nutrient management, particularly ensuring adequate calcium for pod development, is essential for healthy growth, with gypsum often used in calcium-deficient soils [120]. Additionally, maintaining well-drained soils and managing irrigation during key growth stages are critical for maximizing yields and preventing root diseases [121].

## 9. Efficient Water Use and Irrigation Techniques

Water management plays a vital role in peanut cultivation, as the crop is sensitive to both drought and excessive moisture. Ensuring efficient water use is crucial for optimal growth, yield, and sustainability, especially in regions facing water scarcity or irregular rainfall patterns [122]. Peanuts require consistent water availability during critical growth stages such as flowering and pod development, but over-irrigation can lead to root diseases and soil degradation [123]. Implementing efficient irrigation techniques helps peanut farmers to manage water resources more effectively while promoting healthy crop development and maximizing productivity [124]. Drip irrigation, by delivering water directly to peanut plants’ root zones, efficiently reduces water loss from evaporation and runoff, making it an ideal method in drought-prone or water-scarce regions [91]. Thus, by supplying consistent moisture during critical growth periods, such as pegging (when pods begin to form), drip irrigation helps to prevent water stress, which can significantly reduce peanut yields. The use of soil moisture sensors and automated irrigation systems is another important advancement in peanut farming [125]. These technologies enable real-time soil moisture monitoring, ensuring that irrigation is applied only when needed, reducing water wastage, preventing over-irrigation, and optimizing water use for peanut crops [126,127]. Incorporating water-conserving practices like mulching and crop rotation enhances the water-use efficiency in peanut production by retaining soil moisture and improving soil structure [128]. Moreover, a study showed that replacing fallow with forage triticale increased net income by $1016.2 per hectare and improved water use efficiency by 30%, while maintaining economic water use efficiency, making it a recommended option for boosting production, economic returns, and water use efficiency in the nutrient control plan (NCP) region [129]. Thus, it is suggested that the forage triticale–peanut cropping system should be adopted in order to maintain higher forage yield, WUE, and economic benefits in NCP [130]. According to the Peanut Collaborative Research Support Programme (PCRSP), in addition to the multitude of benefits, peanut provides a better nutrient balance for the user [131]. When combined with efficient irrigation, these methods help to conserve water, improve crop health, and support sustainable farming [132].

Case studies and success stories offer valuable insights into improving peanut production through innovative practices and technologies. For example, precision agriculture in the U.S. has enhanced yields and reduced costs by using GPS and variable-rate systems for precise water and fertilizer application [133]. In India, integrating improved peanut varieties with IPM practices has significantly boosted yields and farmer incomes [134]. Brazil’s adoption of drip irrigation and soil moisture sensors has improved the water use efficiency and increased yields in water-scarce regions [135]. Thus, these examples demonstrate the effectiveness of diverse strategies and technologies in optimizing peanut farming and provide valuable lessons for other operations.

## 10. Nutrient Management for Healthy Peanut Plants

Proper nutrient management is essential for ensuring the growth, productivity, and health of peanut plants. Peanuts need a balanced supply of nutrients, including macro and micronutrients, throughout their growth stages, despite being nitrogen-fixing plants [64]. Effective nutrient management optimizes plant growth, prevents deficiencies, and enhances soil fertility for better yields in peanut farming [136]. Phosphorus is especially important for root development and energy transfer, and crucial during early growth and pod-formation stages [137]. Farmers apply phosphorus fertilizers early to support plant growth, as insufficient levels can stunt growth and reduce pod development [138].

Calcium is also crucial for proper pod and seed formation, preventing issues like empty shells or poor seed development [139]. Since calcium is absorbed directly from the soil by the pods, it is important to maintain sufficient calcium levels in the topsoil, especially in sandy soils where calcium may be lacking. Farmers often apply gypsum (calcium sulfate) at the pegging stage to ensure that the plants have access to adequate calcium [140]. Also, effective nitrogen (N) and potassium (K) management in peanut cultivation supports healthy plant growth, improves yield, and enhances resistance to environmental stress [141].

Micronutrients like zinc, boron, copper, molybdenum, and manganese are vital for peanut plant health, supporting enzyme activation, photosynthesis, and root growth [142]. Managing micronutrients is essential for healthy peanut plants, as they support vital physiological processes such as photosynthesis, enzyme function, and nitrogen fixation, ultimately promoting better growth and yield. Therefore, deficiencies in nutrients can result in poor development, reduced disease resistance, and lower yields [143]. Hence, regular soil testing and tailored fertilization strategies help peanut farmers to identify nutrient deficiencies and apply the appropriate fertilizers to maintain optimal nutrient balance, promoting healthy plants and higher yields throughout the growing season.

## 11. Integrated Pest Management (IPM) for Peanuts

IPM in peanut cultivation minimizes pest damage while reducing chemical pesticide use, promoting sustainable crop health and maximizing yields [144]. For example, an assessment study conducted on the economics of groundnut production among smallholder farmers in the Michika local government area of Adamawa State, Nigeria, reported that the government should provide funds that could be used to establish research centers for the development of resistant and improved groundnut seed varieties at a subsidized rate for use by farmers [145]. This can aid in minimizing losses arising from prolonged drought and pests, as well as shortened production period of the crop. IPM combines diverse pest control methods based on ecology and pest biology, enabling farmers to manage pests sustainably through regular monitoring and accurate identification [146]. For peanuts, frequent crop inspections for pests like aphids and leaf spot diseases enable early detection and timely intervention [147]. Integrated monitoring using tools like pheromone traps and field scouting, along with accurate pest identification, ensures effective control strategies, while cultural practices help to reduce pest issues [148]. A review study conducted on cultural practice as an effective pest management approach under integrated pest management summarizes that it is necessary to know about crop and pest biology, ecology, phenology, and their links/interactions to successfully implement cultural practices [149]. For peanuts, this can include practices such as crop rotation, which helps to disrupt the life cycles of pests and diseases by preventing them from becoming established in the soil. According to research conducted on the impact of crop rotation on peanut productivity in rainfed cropping systems, crop rotations that include a year without peanuts in each field consistently lead to higher peanut yields and gross returns compared with monocultures, with residual benefits lasting for two additional peanut crops, significantly reducing peanut pathogens and offering the greatest yield and return advantages in high-yielding seasons [150]. Moreover, according to the groundnut Production Practices Skill Development Series No. 3 ICRISAT Training and Fellowships Program International Crops Research Institute for the Semi-Arid Tropics Patancheru 502 324, Andhra Pradesh, India (1995), a crop rotation of groundnut–cereal–cereal helps in efficient nutrient utilization and reduces soil-borne diseases and nematodes [151]. Research conducted on the crop response to rotation and tillage in peanut-based cropping systems stated that variations in the response to rotation and tillage should be expected based on the crop and edaphic and environmental conditions [152]. Selecting pest-resistant peanut varieties, maintaining soil health, and practicing proper field sanitation help to reduce pest pressure [153]. Chemical control in IPM is used only when necessary, focusing on specific pests while minimizing harm to beneficial organisms and the environment [154]. Combined with biological and physical controls, these methods ensure cost-effective, environmentally responsible pest management for sustainable peanut farming.

## 12. Economic Analysis of Peanut Production

Economic analysis is a critical component in evaluating the viability and profitability of peanut production. For instance, a literature review on the groundnut value chain and market systems in Uganda indicated that target markets for shelled groundnuts were the East African Community (EAC) countries, with potential markets for Ugandan groundnut oil in African nations like Ethiopia, Togo, and Mauritania [155]. As a major cash crop in many regions, peanuts can offer significant financial returns, but its production involves various costs and risks that must be carefully managed [156]. Understanding economic factors helps farmers to optimize production practices and enhance profitability by evaluating costs and potential revenues. Major expenses in peanut farming include land preparation, seeds, fertilizers, pesticides, irrigation, and labor [157]. Irrigation systems are a crucial investment for optimal growing conditions, while labor costs for planting, maintenance, and harvesting also add to expenses. Therefore, analyzing these costs helps farmers to budget effectively and find ways to reduce expenses or increase efficiency [158]. Revenue depends on yield and market prices, which are influenced by factors like soil quality, weather, and pest management [159]. Market prices fluctuate based on supply, demand, and competition, so staying informed about trends is essential for strategic selling [160]. Hence, profitability is calculated by subtracting production costs from revenue, and farmers must manage financial risks, such as price volatility and climate variability [161]. Also, risk-management strategies like crop insurance and forward contracts can help to mitigate these risks and support long-term sustainability [162].

## 13. Technological Innovations in Peanut Farming

Technological innovations have transformed peanut farming by boosting productivity, efficiency, and sustainability. As global demand rises, farmers are adopting advanced technologies to tackle challenges like disease management and resource optimization [163]. Precision agriculture tools using global positioning system (GPS) and remote sensing allow for precise application of inputs, thus reducing waste and improving crop health [164]. Advanced breeding techniques, including genetic modification and marker-assisted selection, develop high-yielding, disease-resistant peanut varieties [165]. Automation and robotics streamline planting, harvesting, and processing, enhancing labor efficiency and reducing costs [166]. These innovations collectively improve productivity while supporting more sustainable and environmentally friendly farming practices.

## 14. Future Line of Works and Perspectives

Future research in peanut production should prioritize developing climate-resilient peanut varieties through advanced genetic techniques, such as gene editing and genomic data integration. This focus will help to create varieties that can withstand changing environmental conditions, like drought and heat. Concurrently, optimizing management practices is essential, with a focus on improving soil management, water use efficiency, and integrated pest management. The use of precision agriculture technologies can aid in modifying these practices for specific field conditions, enhancing productivity and sustainability. Additionally, research should address the socio-economic impacts of these advancements. Considering the economic implications of new technologies and management practices is crucial for their adoption. Investigating the benefits for rural communities, including job creation and economic development, will ensure that innovations in peanut production contribute to broader socio-economic improvements. This integrated approach will help to meet global food security challenges and support the long-term resilience of peanut farming.

## 15. Conclusions and Recommendations

In conclusion, optimizing peanut production pivots on integrating genetic advancements with improved management practices. Advanced genetic techniques are one of the possibilities for adapting peanut varieties to changing environmental conditions and ensuring stable yields, but they are not essential techniques. Employing these genetic innovations will enhance productivity and sustainability by creating peanuts that can withstand stresses like drought and heat. Groundnuts are highly valued among legumes for their rich nutritional profile, including high protein and healthy fat contents, making them a significant dietary staple in many regions. Additionally, their ability to fix atmospheric nitrogen enhances soil fertility, promoting sustainable agricultural practices and benefiting subsequent crops in rotation. Equally important is refining management practices to support healthier crops and higher yields. Modern soil management, advanced irrigation techniques, and integrated pest management are crucial for maximizing production. Precision agriculture technologies can further enhance these practices by providing real-time data for more effective interventions. Finally, understanding the socio-economic impacts of these advancements is also key for widespread adoption.

## Figures and Tables

**Table 2:** Characterization of some of the widely cultivated peanut cultivars globally.

Peanut Cultivars	Characterization of the Known and Widely Cultivated Peanut Crop Cultivars in the World.							
	Altitude Range (masl)	Disease Resistance (%)	Oil Content (%)	Aflatoxin Levels (%)	Carbon Sequestration Potential	N-Fixation Potential	Average Yield (tons)	Ref.
ICGV 91114 (India)	1000	60–70	48–50	0.1–0.5	15–20%	30–50%	1.8 to 2.5	[60]
Tifguard (USA)	1200	90–95	50–52	0.05–0.2	18–22%	30–50%	2.5 to 3.5	[61]
Nyanda (Zimbabwe)	600–1200	75–80	45–50	0.2–0.5	10–15%	40–60%	1.5 to 2.0	[62]
Sedi (Ethiopia)	1200–2000	80–85	50–52	0.1–0.3	12–18%	30–50%	2.0 to 2.5	[63]
Virginia Jumbo (Spain, Greece)	1200	50–60	45–48	0.3–0.6	10–15%	30–50%	2.0 to 3.0	[64]
Florispan (France, Italy)	1000	60–70	46–48	0.2–0.5	12–16%	30–50%	2.0 to 3.0	[65]
Runner (USA)	1000	50–60	48–50	0.1–0.3	10–15%	40–60%	3.0 to 4.5	[66]
TifNV-High O/L (USA)	1000	80–85	42–55	0.05–0.2	12–16%	40–60%	3.0 to 4.5	[67]

**Table 3:** Some of the peanut varieties that are resistant to climate change and resilient, along with their respective developers.

No.	Climate Change Resistant Peanut Varieties	Developer	Range of the Climatic Zone	Ref.
1	ICGV 86590	ICRISAT	Drought tolerance and adaptability to varying climates.	[107]
2	ICGV 93227	ICRISAT	Drought resistance and yield stability under water-limited conditions.	[90]
3	Tifguard	Georgia University (USA)	Tolerate a range of environmental conditions.	[108,109]
4	Shulamit	Israeli Research Institutions	Drought resistance and adaptability to arid conditions.	[91]
5	Huayu 25	Chinese Research Institutions	Resistant to strong drought and high temperatures.	[110]
6	Kashmir Peanuts	Indian Research Institutions	Resilience to cold temperatures.	[111]
7	SPAN 120	Spanish Research Institutions	Drought tolerance and resilience to high temperatures.	[112]
8	Rasi	Indian Research Institutions	Resistance to both drought and high temperatures (Semi-arid).	[77]
9	Ganhua 5	Chinese Research Institutions	Tolerant to drought and heat stress, as well as resistance to common fungal diseases.	[113]
10	G20	Indian Research Institutions	Drought- and heat-resistant.	[91,114]

## Data Availability

Data sharing is not applicable to this article as no new data were analyzed in this study.
